# Demographic and Clinical Characteristics in Different Motor Subtypes of Parkinson’s Disease: How Well Do the Findings Fit Within the Framework of Existing Hypotheses?

**DOI:** 10.3390/neurolint17040051

**Published:** 2025-03-27

**Authors:** Timotej Petrijan, Marija Menih

**Affiliations:** Department of Neurology, University Medical Center Maribor, 2000 Maribor, Slovenia; marija.menih@gmail.com

**Keywords:** Parkinson’s disease, non-motor symptoms, motor subtypes, classification

## Abstract

Background and purpose: This study aimed to investigate risk factors, prodromal signs, and non-motor symptoms (NMSs) across various motor subtypes of Parkinson’s disease (PD) and to interpret the findings within the context of existing hypotheses on PD subtypes. Methods: A search of the database yielded 300 individuals who satisfied the study’s inclusion and exclusion criteria. Among them, 168 patients diagnosed with idiopathic PD underwent a comprehensive evaluation of both motor and non-motor symptoms. The classification of motor subtypes was conducted according to the methodology proposed by Stebbins. Results: The study population consisted of 59.9% males, with an average age of disease onset at 65.45 years. Among them, 87 (51.8%) were classified as having the tremor-dominant (TD) subtype, 61 (36.3%) had the postural instability and gait disorder (PIGD) subtype, and 20 (11.9%) fell into the intermediate (I) subtype. Significant differences between motor subtypes were observed in age at assessment (*p* = 0.03), age at onset (*p* = 0.02), education level (*p* = 0.015), handedness (*p* = 0.013), proportion of non-smokers (*p* = 0.021), cognitive impairment (*p* = 0.003), and apathy (*p* = 0.003). Additionally, statistically significant variations were found across different rating scales and questionnaires, including MoCA (*p* = 0.009), HAM-A (*p* = 0.008), HAM-D (*p* = 0.007), H&Y (*p* = 0.004), SAS (*p* = 0.004), NMSS Domain 3 (mood/apathy) (*p* = 0.003), and NMSS Domain 5 (attention/memory) (*p* = 0.003). Conclusions: The study revealed substantial differences between motor subtypes, underscoring the complexity of PD. These findings highlight the importance of comprehensive evaluations of both MS and NMSs to optimize patient care, improve quality of life, and fit well within the framework of the existing hypotheses of PD subtypes.

## 1. Introduction

Parkinson’s disease (PD), the second most common neurodegenerative disorder, affects over 3% of individuals aged 70 and older [1]. Its diagnosis is based on clinical criteria primarily centered on the identification of motor symptoms and signs (MS). However, non-motor symptoms (NMSs) play a crucial role in the clinical presentation and were included as supportive and exclusion criteria for PD, as well as red flag signs (2). Certain NMSs, such as rapid eye movement sleep behavior disorder (RBD), depression, constipation, and impaired sense of smell, can emerge years before the onset of MS. As the disease progresses, NMSs may become the dominant symptoms and represent the primary burden of the disease [2].

Longitudinal studies have identified several risk factors for PD [3]. Among the most significant are age and gender [4,5]. Additional risk factors that have been validated in at least two prospective studies or meta-analyses, include regular occupational exposure to pesticides and solvents, lack of caffeine consumption, non-smoking status, and genetic predisposition [6].

Identifying subtypes has been recognized as a key priority in PD clinical research [7]. Determining PD subtypes is crucial for uncovering the underlying causes and gaining insight into the progression of the disease. Moreover, subtype identification holds significant potential for informing the design of clinical trials and advancing personalized treatment approaches for PD [8]. The subtypes are not merely different stages of disease progression [9]. The diverse clinical presentation of MS and NMSs indicates that subtypes arise, at least in part, due to varying degrees of Lewy body accumulation and the resulting degeneration in distinct areas of the central and peripheral nervous systems [10]. Beyond the dopaminergic system, multiple other neurotransmitter systems are also implicated, and the combination of dysfunction in multiple neurotransmitter systems is associated with the expression of distinct subtypes [11]. Notably, non-dopaminergic areas may be affected prior to dopaminergic regions [10].

Predefined subtypes based on specific hypotheses are categorized according to predetermined criteria, such as age at onset or MS distribution [8]. Among the various empirical subtypes identified in research and clinical settings, motor subtypes are the most commonly recognized. Two motor subtypes, tremor dominant (TD) and akinetic–rigid, are well-documented in the literature [8]. The introduction of the Movement Disorder Society’s Unified Parkinson’s Disease Rating Scale (MDS-UPDRS) enabled Stebbins et al. [12] to validate motor subtypes, optimizing specificity and sensitivity by utilizing the TD/PIGD ratio to distinguish between tremor-dominant (TD), intermediate (I), and postural instability gait disorder (PIGD) subtypes.

Numerous studies have explored the clinical characteristics, biomarkers, and outcomes associated with motor subtypes. The PIGD subtype is often linked to a higher prevalence of NMSs [13]. The PIGD subtype is particularly associated with an increased risk of apathy [14], as well as sexual dysfunction [15]. In contrast, the TD subtype has been linked to hyposmia [16]. Regarding prognosis, the TD subtype is associated with a more favorable outcome compared to the PIGD subtype [17]. Specifically, the PIGD subtype is linked to a faster rate of cognitive decline, a greater risk of dementia development [18], and an increased propensity for falls [19].

Various biomarkers have been explored across different motor subtypes. The PIGD subtype is notably associated with significant deficits in non-dopaminergic neurotransmission [20]. In terms of imaging markers, this subtype is distinguished by white matter damage in the cortico-limbic regions and medial thalamic areas, along with more pronounced frontal lobe grey matter atrophy [20], while the TD phenotype is associated with reduced cerebellar grey matter volume [21]. Furthermore, plasma neurofilament light chain has been identified as a promising biomarker for both diagnosis and prognosis in early-stage PD patients with the PIGD subtype [22].

Recognizing the particular clinical and demographic characteristics associated with particular motor subtypes can help clinicians anticipate and manage these symptoms more efficiently. This study aimed to investigate prodromal symptoms, risk factors, and NMSs across different motor subtypes while contextualizing the findings within established hypotheses on PD phenotypes.

## 2. Materials and Methods

Patients diagnosed with idiopathic PD and assessed at the University Department of Neurology, University Medical Centre Maribor between 2013 and 2023 were enrolled in an ethically approved study (Slovenian National Medical Ethics Committee, No. 0120-509/2019/4).

Eligibility for inclusion required a diagnosis of idiopathic PD based on the UK Parkinson’s Disease Society Brain Bank Criteria [23], an age of 18 years or older, and disease severity classified within H&Y stages 1–5. Exclusion criteria encompassed the presence of secondary or atypical parkinsonism, significant cognitive impairment (MoCA < 20), or major psychiatric disorders.

Demographic and clinical characteristics were recorded, including sex, handedness, educational attainment (following the International Standard Classification of Education—ISCED), age at examination, disease onset age, disease duration (<5 years, 5–10 years, >10 years), exposure to PD risk factors, prevalence and chronology of NMSs, and initial prodromal symptoms. Data collection adhered to the Movement Disorder Society (MDS) Research Criteria [6].

The study incorporated established risk factors validated by at least two prospective cohort studies or meta-analyses, including male sex, occupational or frequent non-occupational exposure to pesticides (≥100 instances), minimal caffeine intake (<3 cups per week), occupational solvent exposure, non-smoking status, and a family history of PD. Additional data were collected on risk factors with less conclusive evidence, such as alcohol consumption, rural living, and history of head trauma [6].

Prodromal symptoms considered in the study encompassed RBD, constipation requiring medical intervention more than once per week or bowel movements occurring less than once every two days, impaired olfaction, excessive daytime sleepiness (EDS), sexual dysfunction, symptomatic hypotension, clinically diagnosed depression (with or without anxiety), and urinary disturbances (excluding long-term stress incontinence in women).

Neurological evaluations were conducted by specialists in extrapyramidal disorders, utilizing validated rating scales and questionnaires recommended by the MDS working group for PD patients. NMSs were assessed using the Non-Motor Symptoms Assessment Scale for Parkinson’s Disease (NMSS) [24]. Additional tools included the Montreal Cognitive Assessment (MoCA) [25], Hamilton Depression Scale (HAM-D) [26], Hamilton Anxiety Rating Scale (HAM-A) [27], Epworth Sleepiness Scale (ESS) [28], REM Sleep Behavior Disorder Screening Questionnaire (RBDSQ) [28], Starkstein Apathy Scale (SAS) [29], and Fatigue Severity Scale (FSS) [28].

MS were evaluated using the MDS-UPDRS III and the H&Y scale to determine disease progression [30]. All assessments were performed while patients were in the “on” phase of their medication regimen. The levodopa equivalent daily dose was calculated based on the Tomlinson et al. method [31]. Classification of motor subtypes followed the methodology described by Stebbins [12]. Only NMSs persisting for at least three months were included in the analysis, and structured interviews were conducted in the presence of a relative or caregiver to enhance accuracy.

### Statistical Analysis

Statistical analyses were performed using the open-source software Jamovi v2.3 alongside the SciPy Python library v1.11. The normality of data distribution was evaluated using the Shapiro–Wilk test. Depending on the results, appropriate parametric or non-parametric tests were applied. A significance threshold of 5% (*p* ≤ 0.05) was used to determine statistical significance, leading to the rejection of the null hypothesis when this criterion was met. To control for the false discovery rate (FDR) in multiple hypothesis testing, the Benjamini–Hochberg procedure was implemented with an FDR threshold of 0.05. Comparisons of demographic and clinical variables among the three motor subtypes were conducted using ANOVA, Kruskal–Wallis, or chi-squared tests, based on variable characteristics. For post hoc analysis following Kruskal–Wallis tests, the Dwass–Steel–Critchlow–Fligner method was employed for pairwise comparisons. Additionally, post hoc analyses for chi-squared independence tests were conducted using z-scores of adjusted standardized residuals. To correct for multiple comparisons, Bonferroni adjustments were applied to all *p*-values obtained from post hoc tests.

## 3. Results

### Demographic Trends and Clinical Features

A database screening initially identified 300 individuals who met the study’s inclusion and exclusion criteria. Among them, 204 patients agreed to participate and provided written informed consent. The final analysis included 168 patients who successfully completed the baseline assessments for motor and non-motor symptoms.

Table 1 outlines the demographic and clinical characteristics of the entire PD cohort, as well as the three distinct motor subtypes. Of the participants, 59.9% were male, with a mean age of 71.7 years and an average disease onset at 65.45 years. Regarding disease duration, 45.2% had been diagnosed for less than five years, while 79.8% had a disease duration of less than ten years. Furthermore, 85.7% of the participants were right-handed.

Educational background analysis revealed that 73.2% had completed at least secondary education (corresponding to level 3 or higher based on ISCED classification). A family history of PD was reported in 16.1% of the cohort. The distribution of motor subtypes included 87 patients (51.8%) classified as tremor-dominant (TD), 61 patients (36.3%) as postural instability gait disorder (PIGD), and 20 patients (11.9%) as intermediate (I).

**Figure 1:**
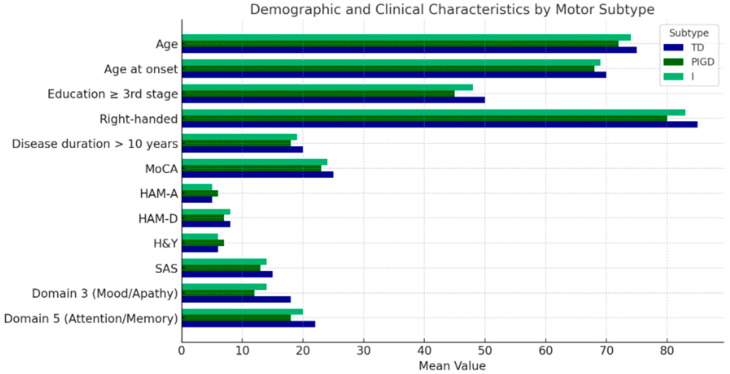
A visual depiction of the demographic and clinical traits of study participants, categorized by motor subtypes: tremor dominant (TD) (blue), postural instability gait disorder (PIGD) (dark green), and intermediate (I) (green).

Across motor subtypes, significant differences were observed in demographic and clinical characteristics, including age at evaluation (*p* = 0.03), age at disease onset (*p* = 0.02), education level (*p* = 0.015), and handedness (*p* = 0.013) (Table 1, Figure 1). Additionally, motor subtypes varied in the proportion of non-smokers (*p* = 0.021) (Table 2).

At the individual symptom level, significant differences were identified in cognitive impairment (*p* = 0.003) and apathy (*p* = 0.003) (Table 3). Prevalence of initial NMSs in the total study cohort and across motor subtypes is presented in Table 4.

Statistically significant variations were also found across multiple rating scales and questionnaires, including MoCA (*p* = 0.009), HAM-A (*p* = 0.008), HAM-D (*p* = 0.007), H&Y (*p* = 0.004), SAS (*p* = 0.004), NMSS Domain 3 (Mood/Apathy) (*p* = 0.003), and NMSS Domain 5 (Attention/Memory) (*p* = 0.003) (Table 1, Figure 1).

We further divided the patients into three subgroups based on disease duration: <5 years, 5–10 years, and >10 years. The distribution of each motor subtype within these groups is shown in Table 5. Through subanalysis, we confirmed that differences in demographic and clinical characteristics among motor subtypes persist even within each disease duration group. The *p*-values are presented in Table 6.

## 4. Discussion

The findings from our study reveal significant insights into the demographic and clinical characteristics associated with different motor subtypes of PD. These subtypes not only exhibit distinct MS but also differ markedly in their burden of NMSs, cognitive function, and overall disease progression. This discussion will contextualize our findings within existing hypotheses regarding PD phenotypes.

From studies based on cluster analysis, the most consistent subtypes identified are the diffuse malignant subtypes, characterized by a wide range of NMSs and MS along with a poorer prognosis. Those subtypes are marked by postural instability and gait difficulty, older age at onset, a higher burden of NMSs, particularly cognitive function disturbances, and a more rapid progression to milestones such as dementia, reduced mobility, institutionalization, and death. Patients with the diffuse malignant subtypes experience a significant burden of NMSs even in the early stages of the disease, especially concerning RBD and other sleep disturbances, dysautonomia, and cognitive impairments [32,33,34]. Those subtypes align well with the PIGD subtype in our study, which was characterized by an older age at diagnosis, lower MoCA scores, and higher SAS, H&Y, Domain 3 (Mood/Apathy), and Domain 5 (Attention/Memory) NMSS scores. The PIGD subtype was also associated with a lower level of education. Among all three motor subtypes, patients with the PIGD subtype more often reported a history of mild head injury, olfactory dysfunction, and EDS. RBD was present in more than 30% of patients. However, the differences were not statistically significant.

Significant cognitive impairment and apathy among PIGD patients in our study further support the notion that this subtype is associated with a more substantial overall burden of disease. Analyses of cerebrospinal fluid have demonstrated that individuals with diffuse malignant subtypes are more likely to present a cerebrospinal fluid profile similar to Alzheimer’s disease (AD), characterized by reduced β-amyloid levels and elevated tau concentrations [35]. Neuropathological investigations further reveal that patients with REM sleep behavior disorder or diffuse malignant subtypes exhibit a higher accumulation of α-synuclein deposits in both the brain and the gut compared to those with other Parkinson’s disease subtypes [34,36,37]. Notably, in our study, patients with the PIGD subtype were less frequently right-handed compared to those with the TD and I subtypes. Handedness has been associated with Alzheimer’s disease (AD), with some research suggesting that left-handedness or related traits may contribute to earlier cognitive decline in AD [38]. One study reported a higher prevalence of left-handedness in early-onset AD cases compared to those with late-onset AD [39]. Genetics play a key role in determining hand preference, and the same genetic factors that influence handedness have also been implicated in both PD and AD. Additionally, research suggests a link between the brain development processes associated with handedness and an individual’s risk of developing PD [40]. Olfactory dysfunction in PD has also been correlated with poorer cognitive performance and may serve as an early predictor of dementia in PD. A longitudinal study by Baba et al. [41] identified severe olfactory impairment as a potential prodromal marker for PD-related dementia. Interestingly, olfactory dysfunction has been associated with cholinergic system impairment, which aligns with findings that hyposmia does not improve with levodopa treatment. On the other hand, rasagiline has been reported to significantly enhance odor discrimination in patients with early-stage PD [42]. Olfactory dysfunction tends to be more severe in the PIGD motor phenotype [42]. The abovementioned observations were also confirmed in our patients with the PIGD subtype. These patients may not only present differently but also respond differently to treatment.

In contrast, the motor benign subtypes, characterized by a younger age at onset, tremor, a lower burden of NMSs, and a slower progression, reflect the opposite end of the spectrum. Patients with those subtypes typically experience a more favorable prognosis [9,43,44]. These subtypes align well with the TD subtype in our study characterized by younger age at onset, higher MoCA scores, and lower H&Y scores. The TD subtype was also associated with a higher level of education. Among all three motor subtypes, patients with the TD subtype more often reported alcohol and caffeine consumption and depression. However, the differences were not statistically significant.

Long-term alcohol consumption has been linked to structural alterations and changes in neural connectivity even in individuals without neurological conditions [45]. Among the most affected brainstem structures are the raphe nuclei, which are crucial for serotonin production—a neurotransmitter that plays a fundamental role in mood regulation. Patients with the TD subtype in our study had significantly higher scores on the HAM-A and HAM-D scales, which assess anxiety and depressive symptoms in patients with PD.

Our study also found a significantly higher proportion of smokers among patients with the TD subtype. This association may be influenced by various factors, including genetic predispositions, lifestyle choices, and the neuroprotective effects of nicotine. Smoking has an undefined biological neuroprotective effect on the development of PD [46]. One possible explanation for the higher prevalence of smoking in TD patients is the neuroprotective effects of nicotine. Nicotine has been shown to have dopaminergic effects, which may help mitigate some of the MS associated with PD. Studies have suggested that nicotine may enhance dopaminergic transmission and reduce the risk of developing PD [47]. Consequently, individuals with a predisposition to the TD subtype may be more likely to engage in smoking as a means of self-medication to alleviate their symptoms. Psychological factors may also play a role in the higher prevalence of smoking among TD patients. Research has shown that individuals with PD often experience anxiety and depression, which can lead to increased smoking as a coping mechanism [48]. The prevalence of anxiety and depression was the highest in the TD subtype in our study. However, the difference did not reach statistical significance.

The early pathological accumulation and dissemination of α-synuclein in Parkinson’s disease (PD) may originate in either the central or peripheral nervous system. One prevailing hypothesis suggests that PD consists of two primary subtypes, each differing in its initial site of pathology and progression mechanisms—body-first or bottom-up and brain-first or top-down—provides a compelling framework for understanding the differences observed in our study. The body-first subtype, in which α-synuclein pathology is believed to originate in the autonomic and enteric nervous systems before spreading to the central nervous system via the vagus nerve and sympathetic pathways, may account for the presence of dysautonomia preceding MS in patients with the TD and I subtypes [9,43,44]. However, the difference in the prevalence of dysautonomia among all three subtypes was not statistically significant in our study. Conversely, the brain-first subtype, where pathology hypothetically arises directly in the brain or through entry into the olfactory system and progresses through the brainstem to the peripheral nervous system, may account for the cognitive and smell disorders as prodromes more commonly seen in PIGD patients [9,43,44,49].

These subtypes correspond closely to findings from postmortem studies, which have identified two main patterns of Lewy body pathology. The brainstem-dominant pattern of α-synuclein accumulation is thought to align with the body-first subtype, while the limbic-dominant pattern is more consistent with the brain-first subtype [50,51]. If validated, this hypothesis could have major implications for our understanding of Lewy body disorders. Moreover, specific triggering factors, such as microbiome alterations and leaky gut syndrome, may contribute to the development of the body-first subtype [52,53].

Biological markers relevant for prodromal diagnosis and monitoring progression would likely depend on the subtype. For example, heart scintigraphy with MIBG (meta-iodobenzylguanidine) is not a definitive tool for ruling out prodromal PD, as most individuals with the brain-first subtype initially exhibit normal scintigraphy results. In contrast, MIBG scintigraphy is more effective for monitoring the progression of the body-first subtype, as cardiac denervation may precede the degeneration of the nigrostriatal dopaminergic system by five to fifteen years [54].

The development of therapeutic agents with the potential to simultaneously alleviate both MS and NMSs by targeting multiple affected neurotransmission systems in the central nervous system represents a highly promising approach for managing this severely disabling disorder. Given the broad range of dynamic changes occurring in various neurotransmitter systems in PD-affected brains, it appears that the future of PD therapy will be directed toward multi-therapeutic strategies. These strategies will be tailored to specific subtypes and the temporal evolution of symptoms in individual patients, enabling more targeted and effective treatment of both MS and NMSs [55].

Extensive research has linked cognitive impairment primarily to dysfunction in the cholinergic system, with secondary contributions from the dopaminergic, glutamatergic, and purinergic systems [55]. In PD dementia, the reduction in choline acetyltransferase activity is even more pronounced than in AD. The administration of anticholinergic therapy in PD patients significantly worsens memory function, indicating an underlying subclinical cholinergic deficit in PD. Conversely, rivastigmine, an acetylcholinesterase and butyrylcholinesterase inhibitor, has been shown to improve cognitive function in PD patients [55]. As we mentioned above, rasagiline has also been reported to significantly enhance odor discrimination in PD patients, which was more pronounced in PIGD subtype in our study [43]. Whether degeneration of the cholinergic basal forebrain is directly responsible for cognitive decline or whether the relationship is mediated through closely interconnected brain structures, such as those in the medial temporal lobe, remains unclear. A study by Berlot et al. [56] suggests that the integrity of the basal forebrain is correlated with hippocampal volume. Additionally, there is a known relationship between visuospatial functions and cholinergic nuclei, but this connection appears to be entirely mediated by hippocampal structural variations. Gait and balance impairments are major causes of falls, significantly affecting quality of life, functional status, morbidity, and mortality in PD patients [57]. Falls are directly associated with cholinergic system dysfunction in the pedunculopontine nucleus (PPN) and basal nucleus of Meynert, independent of dopamine loss [58]. Those observations suggest that targeted treatment of the PIGD subtype could involve pharmacological agents specifically modulating the cholinergic system. However, the role of anticholinergic drugs must be carefully considered. These drugs have been shown to improve PD clinical manifestations, with researchers recognizing their effectiveness as monotherapy or in combination with dopaminergic agents [59]. Their widespread use reflects their versatile therapeutic value in PD treatment strategies [57].

Depression and anxiety, which were more frequently reported in patients with the TD subtype, are characterized by predominant dysfunction in the serotonergic system, with secondary involvement of the noradrenergic, glutamatergic, and GABAergic systems [55]. Additionally, in the context of autonomic dysfunction, which was also more frequently reported in TD patients, serotonergic and noradrenergic system abnormalities are the primary contributors [55]. For the management of the TD subtype, modulating these neurotransmitter systems may be a promising approach, targeting the specific pathophysiological mechanisms that contribute to the development and persistence of these symptoms.

These findings further highlight the growing interest in targeting neurotransmitter systems beyond the dopaminergic system to address both MS and NMSs in PD. The inclusion of NMDA receptor antagonists, AMPA receptor modulators, mGlu1 and mGlu5 inhibitors, and other agents in the therapeutic arsenal for PD not only opens new possibilities for symptom relief but also offers potential for slowing or preventing neurodegenerative progression. This approach supports the idea that a broader spectrum of therapeutic strategies is required, moving beyond the traditional focus on dopaminergic modulation. By addressing the complexity of PD and the diverse symptomatology experienced by patients, such interventions offer hope for a more comprehensive management approach that considers both disease pathology and patient-specific symptom burdens [57].

In our study, we observed a notable prevalence of alcohol and caffeine consumption, as well as a higher rate of smoking among patients with the TD subtype. This raises important considerations regarding the incorporation of non-pharmacological interventions into the treatment paradigm tailored to specific motor subtypes. Emerging evidence supports the efficacy of exercise as a fundamental non-pharmacological intervention for managing both MS and NMSs. Research has shown that structured exercise programs can significantly improve mobility, balance, and overall quality of life [60]. For the TD subtype, characterized by tremors and often accompanied by symptoms of anxiety and depression, regular physical activity can serve as an essential strategy to mitigate these NMSs, which are frequently as debilitating as the motor manifestations [61]. Additionally, engaging in aerobic activities or strength training can have neuroprotective effects, potentially slowing disease progression and enhancing motor function through neuroplastic changes [62]. Furthermore, specific forms of exercise, such as Nordic walking or dancing, have gained recognition for their dual impact on both motor function and psychosocial well-being. These activities not only improve gait and reduce fall risk but also foster social interactions, which can be beneficial for combating the feelings of isolation often associated with PD [63]. Integrating these interventions as part of a comprehensive management plan may enhance adherence to treatment and lead to better patient outcomes. Occupational therapy and speech therapy also play critical roles in addressing the functional challenges faced by PD patients, particularly in those exhibiting higher levels of anxiety or cognitive decline. Tailoring these therapeutic interventions to the individual needs associated with each motor subtype can empower patients and improve their functionality [64]. For instance, cognitive–behavioral strategies specifically designed to address the anxieties linked with the TD subtype can significantly improve psychological outcomes, thereby enriching overall quality of life [61]. Moreover, lifestyle modifications, such as diet and smoking cessation, should be actively encouraged among patients, especially those in the TD subtype, given the association observed with lifestyle factors. Evidence suggests that regular physical activity and a balanced diet may not only modulate symptom severity but could also improve long-term prognosis in patients with PD [65]. For those who consume alcohol, understanding the potential risks and benefits associated with moderation can guide individualized care pathways [65].

Our study has certain limitations that should be acknowledged. First, the patient cohort may not fully represent the general population, as the data were obtained from a single university center, potentially limiting the generalizability of the findings. The findings may not be generalizable to more ethnically diverse populations or larger cohorts. Additionally, a longitudinal study design would be more appropriate for evaluating the prognostic value of individual variables over time.

Second, the ideal candidates for studying PD subtypes would be untreated patients, as dopaminergic therapy may influence the presentation of NMSs. However, due to ethical considerations, the number of untreated patients was limited and the majority of participants in our study were receiving symptomatic therapy. To account for the effects of treatment, we calculated the equivalent daily doses of levodopa for all dopaminergic therapies administered to participants.

Third, the heterogeneity of PD presents challenges in subtyping, particularly due to the overlap between different subtypes and the instability of these classifications. Evaluating individual motor and non-motor symptoms can be challenging, particularly due to fluctuations influenced by intrinsic compensatory mechanisms, medication effects, and the natural progression of the disease. To standardize our assessments, all patients were tested during the “on” phase of their medication. However, evaluating patients in the “off” would also be beneficial, as this could provide a more comprehensive understanding of symptom fluctuations. The subjective nature of NMSs and the frequent lack of patient insight complicate identifying and quantifying these fluctuations. While the methodology and statistical analyses have been conducted rigorously, we acknowledge the limitations of the Stebbins’ classification method utilized in this analysis. The temporal variability of MS presents a challenge, with initial classifications of TD patients potentially shifting over time as their disease progresses. However, we opted for this classification framework to align our study with prevailing academic discourse surrounding motor subtypes, recognizing that it primarily emphasizes motor function while potentially overlooking the varying responses to pharmacological treatment [34,66]. This choice underlines our intention to focus on motor symptomatology while suggesting that future work could indeed benefit from encompassing a wider range of contributing factors, including neurogenetic underpinnings [36].

As we mentioned, one of the challenges in phenotyping is the potential change in motor subtype in individual patients as the disease progresses (phenotypic conversion). Studies on PD phenotyping typically include individuals at various disease stages and analyze cohorts without stratification by disease duration or severity. As a result, phenotypic changes over time are not accounted for. For example, motor subtypes tend to be more heterogeneous in the early stages of the disease but gradually converge into a common subtype toward the later stages [67]. Classification systems thus represent an attempt to simplify the main disease subtypes, but in reality, these subtypes often overlap. To address this issue, we divided patients into three groups based on disease duration: <5 years, 5–10 years, and >10 years. However, we acknowledge that a longitudinal study would be more appropriate to confirm or refute the hypothesis of phenotypic conversion. We demonstrated that differences in demographic and clinical characteristics among motor subtypes persist even within each disease duration group, which could argue against the hypothesis of phenotypic conversion. Across all three disease duration groups, the TD subtype was the most prevalent, followed by the PIGD and I subtypes.

Lastly, there is a limited amount of research investigating the connection between clinically defined subtypes and the underlying biological mechanisms of the disease. Understanding this relationship is crucial, as it may contribute to developing subtype-targeted therapies.

## 5. Conclusions

In conclusion, while our study provides valuable insights into the PD patients’ demographic and clinical characteristics across different motor subtypes, it is essential to acknowledge its limitations and the need for further research. The alignment of our findings with the existing literature emphasizes the complexity of PD and the importance of a multifaceted approach to patient care. Future studies should continue to explore the interplay between MS and NMSs and the impact of biological markers on disease progression and treatment outcomes. This comprehensive understanding will be essential for enhancing patient care and improving the quality of life for individuals living with PD. The significant differences in NMS prevalence and severity among motor subtypes underscore the importance of a comprehensive assessment that includes both MS and NMSs. As such, clinicians should be aware of the distinct profiles associated with each motor subtype to tailor interventions effectively.

## Figures and Tables

**Table 1:** Clinical and demographic attributes of the study group.

	Overall (*n* = 168) (100%)	TD (*n* = 87) (51.8%)	PIGD (*n* = 61) (36.3%)	I (*n* = 20) (11.9%)	*p*-Value	Adj. *p*-Value *	Post Hoc Statistical Analysis **
Gender (male)	100 (59.9%)	5 (63.2%)	34 (55.7%)	11 (55.0%)	0.599	0.682	
Age (years)	71.70 ± 9.57	68.02 ± 9.63	77.41 ± 7.17	70.25 ± 7.36	0.001 ^KW^	0.030	TD-PIGD, PIGD-I
Age at onset (years)	65.45 ± 10.18	61.34 ± 9.39	71.46 ± 8.79	65.00 ± 8.69	0.001 ^KW^	0.020	TD-PIGD, PIGD-I
Education ≥ 3. stage based on ISCED (%)	123 (73.2%)	76 (82.8%)	34 (55.7%)	13 (65.0%)	<0.001 ^χ2^	0.015	
Right-handed (%)	144 (85.7%)	81 (93.1%)	44 (72.1%)	19 (95.0%)	0.005 ^χ2^	0.013	TD/PIGD-Left/Right
Disease duration > 10 years (%)	34 (20.2%)	19 (21.8%)	12 (19.7%)	3 (15.0%)	<0.001 ^χ2^	0.012	TD, PIGD
Family history	27 (16.1%)	12 (13.8%)	12 (19.7%)	3 (15.0%)	0.233 ^χ2^	0.318	
Side of onset—right (%)	92 (54.8%)	43 (49.4%)	39 (63.9%)	10 (50.0%)	0.196 ^χ2^	0.287	
No. of NMSs	6.88 ± 3.21	7.09 ± 3.56	6.89 ± 2.57	5.95 ± 3.36	<0.001 ^χ2^	0.010	No pair
MoCA	25.77 ± 2.55	26.75 ± 2.11	24.08 ± 2.30	26.70 ± 2.39	<0.001 ^KW^	0.009	TD-PIGD, PIGD-I
HAM-A	6.05 ± 5.32	7.32 ± 5.33	4.79 ± 5.42	4.4 ± 3.39	<0.001 ^KW^	0.008	TD-PIGD
HAM-D	6.95 ± 5.61	8.36 ± 6.16	5.59 ± 5.06	5.00 ± 2.22	<0.001 ^KW^	0.007	TD-PIGD
UPDRS III	37.37 ± 10.96	37.08 ± 10.02	37.62 ± 10.94	37.85 ± 14.93	<0.001 ^KW^	0.006	No pair
H&Y	2.45 ± 0.70	2.15 ± 0.56	2.92 ± 0.46	2.35 ± 0.75	<0.001 ^KW^	0.005	TD-PIGD, PIGD-I
LED	725.00 ± 285.76	693.05 ± 278.51	740.33 ± 271.01	817.25 ± 346.89	<0.001 ^KW^	0.005	No pair
ESS	6.72 ± 4.24	6.54 ± 3.79	7.08 ± 4.38	6.40 ± 5.65	<0.001 ^KW^	0.005	No pair
FSS	31.81 ± 13.18	31.21 ± 13.46	32.25 ± 12.02	33.10 ± 15.71	<0.001 ^KW^	0.004	No pair
RBDSQ	4.92 ± 2.65	5.10 ± 2.56	4.70 ± 2.73	4.80 ± 2.84	<0.001 ^KW^	0.004	No pair
SAS	11.35 ± 6.39	10.05 ± 6.38	14.44 ± 5.48	7.55 ± 5.15	<0.001 ^KW^	0.004	TD-PIGD, PIGD-I
NMSS	59.38 ± 36.94	56.95 ± 37.13	63.34 ± 36.83	57.80 ± 37.25	<0.001 ^KW^	0.004	No pair
Domain 1 (cardiovascular including fall)	3.00 ± 3.91	2.74 ± 3.50	3.13 ± 4.24	3.75 ± 4.66	0.416 ^KW^	0.509	
Domain 2 (sleep/fatigue)	9.89 ± 8.58	9.44 ± 8.28	10.33 ± 9.33	10.55 ± 7.74	<0.001 ^KW^	0.003	No pair
Domain 3 (mood/apathy)	13.44 ± 16.23	17.71 ± 18.48	8.28 ± 11.44	10.60 ± 13.33	<0.001 ^KW^	0.003	TD-PIGD
Domain 4 (perceptual problem/hallucination)	0.57 ± 1.73	0.84 ± 2.11	0.28 ± 1.10	0.30 ± 1.34	<0.001 ^KW^	0.003	No pair
Domain 5 (attention/memory)	11.22 ± 12.21	5.56 ± 8.01	20.08 ± 12.15	8.8 ± 12.16	<0.001 ^KW^	0.003	TD-PIGD, PIGD-I
Domain 6 (gastrointestinal tract)	4.93 ± 6.06	5.05 ± 6.20	4.80 ± 6.27	4.85 ± 4.96	0.074 ^KW^	0.139	
Domain 7 (urinary)	6.30 ± 8.91	5.84 ± 8.28	6.31 ± 9.32	8.30 ± 10.35	0.517 ^KW^	0.620	
Domain 8 (sexual function)	3.82 ± 3.71	4.18 ± 4.22	3.43 ± 2.94	3.45 ± 3.43	0.007 ^KW^	0.017	No pair
Domain 9 (miscellaneous)	6.45 ± 5.96	6.17 ± 5.58	6.59 ± 6.55	7.20 ± 5.85	<0.001 ^KW^	0.003	No pair

* Benjamini–Hochberg correction method applied. ** Statistically significant pairwise comparisons from post hoc analysis. ^χ2^ = Chi-square test for independence, ^KW^ = Kruskal–Wallis test for independent samples. TD: tremor dominant; PIGD: postural instability gait disorder; I: intermediate.

**Table 2:** Risk factors for PD and prodromal symptoms in the overall study population.

Risk Factors						
	Overall (*n* = 168) (100%)	TD (*n* = 87) (51.8%)	PIGD (*n* = 61) (36.3%)	I (*n* = 20) (11.9%)	*p*-Value	Adj. *p*-Value *
Pesticides (%)	53 (31.5%)	26 (29.9%)	20 (32.8%)	7 (35.0%)	0.876 ^χ2^	0.906
Solvents (%)	28 (16.7%)	17 (19.5%)	6 (9.8%)	5 (25.0%)	0.168 ^χ2^	0.258
Rural environment (%)	111 (66.1%)	60 (69.0%)	35 (57.4%)	16 (80.0%)	0.128 ^χ2^	0.226
Head injury (%)	26 (15.5%)	10 (11.5%)	13 (21.3%)	3 (15.0%)	0.266 ^χ2^	0.351
Non-caffeine (%)	56 (33.3%)	26 (29.9%)	23 (37.7%)	7 (35.0%)	0.602 ^χ2^	0.682
Alcohol (%)	53 (31.5%)	35 (40.2%)	12 (19.7%)	6 (30.0%)	0.143 ^χ2^	0.238
Non-smoking (%)	104 (61.9%)	26 (31.0%)	29 (47.5%)	8 (40.0%)	0.009 ^KW^	0.021
RBD (%)	49 (29.2%)	28 (32.2%)	19 (31.1%)	2 (10.0%)	0.132 ^χ2^	0.226
Smell disorder (%)	46 (27.4%)	20 (23.0%)	22 (36.1%)	4 (20.0%)	0.157 ^χ2^	0.248
Constipation (%)	51 (30.4%)	27 (31.0%)	19 (31.1%)	5 (25.0%)	0.857 ^χ2^	0.902
EDS (%)	15 (8.9%)	8 (9.2%)	7 (11.5%)	0 (0.0%)	0.293 ^χ2^	0.374
Hypotension (%)	23 (13.7%)	8 (9.2%)	11 (18.0%)	4 (20.0%)	0.209 ^χ2^	0.292
Sexual dysfunction (%)	29 (17.3%)	15 (17.2%)	10 (16.4%)	4 (20.0%)	0.934 ^χ2^	0.934
Micturition dysfunction (%)	17 (10.1%)	5 (5.7%)	8 (13.1%)	4 (20.0%)	0.101 ^χ2^	0.184
Depression (%)	39 (23.2%)	26 (29.9%)	7 (11.5%)	6 (30.0%)	0.025 ^χ2^	0.056

* Benjamini–Hochberg correction method applied. ^χ2^ = Chi-square test for independence, ^KW^ = Kruskal–Wallis test for independent samples. TD: tremor dominant; PIGD: postural instability gait disorder; I: intermediate.

**Table 3:** Prevalence of individual NMSs in the total study cohort and across motor subtypes.

NMSs	Overall (*n* = 168) (100%)	TD (*n* = 87) (51.8%)	PIGD (*n* = 61) (36.3%)	I (*n* = 20) (11.9%)	*p*-Value *	Adj. *p*-Value (Benjamini-Hochberg)
Salivation (%)	38 (22.6%)	24 (27.6%)	11 (18.0%)	3 (15.0%)	0.269 ^χ2^	0.351
Smell disorder (%)	54 (32.1%)	22 (25.3%)	27 (44.3%)	5 (25.0%)	0.040 ^χ2^	0.086
Dysphagia (%)	36 (21.4%)	20 (23.0%)	13 (21.3%)	3 (15.0%)	0.734 ^χ2^	0.816
Nausea/vomiting (%)	40 (23.8%)	26 (29.9%)	8 (13.1%)	6 (30.0%)	0.049 ^χ2^	0.098
Constipation (%)	75 (44.6%)	41 (47.1%)	26 (42.6%)	8 (40.0%)	0.782 ^χ2^	0.853
Micturition dysfunction (%)	81 (48.2%)	38 (43.7%)	31 (50.8%)	12 (60.0%)	0.369 ^χ2^	0.461
Sexual dysfunction (%)	97 (57.7%)	55 (63.2%)	34 (55.7%)	8 (40.0%)	0.153 ^χ2^	0.248
Dizziness (%)	94 (56.0%)	47 (54.0%)	36 (59.0%)	11 (55.0%)	0.831 ^χ2^	0.890
Sleep dysfunction (%)	121 (72.0%)	63 (72.4%)	44 (72.1%)	14 (70.0%)	0.976 ^χ2^	0.945
Legs swelling (%)	40 (23.8%)	21 (24.1%)	14 (23.0%)	5 (25.0%)	0.977 ^χ2^	0.945
Excessive sweating (%)	48 (28.6%)	32 (36.8%)	13 (22.3%)	3 (15.0%)	0.044 ^χ2^	0.091
Diplopia (%)	21 (12.5%)	11 (12.6%)	8 (13.1%)	2 (10.0%)	0.934 ^χ2^	0.934
Pain (%)	86 (51.2%)	48 (55.2%)	29 (47.5%)	9 (45.0%)	0.553 ^χ2^	0.651
Depression (%)	91 (54.2%)	55 (63.2%)	27 (44.3%)	9 (45.0%)	0.051 ^χ2^	0.099
Anxiety (%)	78 (46.4%)	46 (52.9%)	23 (37.7%)	9 (45.0%)	0.189 ^χ2^	0.284
Psychosis (%)	27 (16.1%)	18 (20.7%)	6 (9.8%)	3 (15.0%)	0.207 ^χ2^	0.292
Cognitive impairment (%)	102 (60.7%)	41 (47.1%)	51 (83.6%)	10 (50.0%)	<0.001 ^χ2^	0.003
Apathy (%)	53 (31.5%)	20 (23.0%)	31 (50.8%)	2 (10.0%)	<0.001 ^χ2^	0.003

* All hypotheses were tested using ^χ2^ test of independence.

**Table 4:** Prevalence of initial NMSs in the total study cohort and across motor subtypes.

First NMS	Overall (*n* = 168) (100%)	TD (*n* = 87) (51.8%)	PIGD (*n* = 61) (36.3%)	I (*n* = 20) (11.9%)
Smell disorder (%)	32 (19.0%)	12 (13.8%)	16 (26.2%)	4 (20.0%)
Constipation (%)	33 (19.6%)	17 (19.5%)	13 (21.3%)	3 (15.0%)
Micturition dysfunction (%)	2 (1.2%)	1 (1.1%)	0	1 (5.0%)
Sexual dysfunction (%)	8 (4.8%)	1 (1.1%)	3 (4.9%)	4 (20.0%)
Dizziness (%)	9 (5.4%)	3 (3.4%)	5 (8.2%)	1 (5.0%)
Sleep dysfunction (%)	27 (16.1%)	15 (17.2%)	10 (16.4%)	2 (10.0%)
Legs swelling (%)	1 (0.6%)	1 (1.1%)	0	0
Excessive sweating (%)	10 (6.0%)	5 (5.7%)	3 (4.9%)	2 (10.0%)
Pain (%)	9 (5.4%)	6 (6.9%)	2 (3.3%)	1 (5.0%)
Depression (%)	19 (11.3%)	15 (17.2%)	2 (3.3%)	2 (10.0%)
Anxiety (%)	13 (7.7%)	11 (12.6%)	2 (3.3%)	0
Cognitive impairment (%)	4 (2.4%)	0	4 (6.6%)	0

**Table 5:** Frequencies of each motor subtype within disease duration groups.

Disease Duration		N	Percentage (%)
<5 years	TD	36	47.4%
	PIGD	30	39.5%
	I	10	13.2%
	Total	76	100.0%
5–10 years	TD	32	55.2%
	PIGD	19	32.8%
	I	7	12.1%
	Total	58	100.0%
>10 years	TD	19	55.9%
	PIGD	12	35.3%
	I	3	8.8%
	Total	34	100.0%

TD: tremor dominant; PIGD: postural instability gait disorder; I: intermediate.

**Table 6:** Statistical test *p*-values for differences in demographic and clinical characteristics of motor subtypes based on disease duration.

	Age	Age at Onset	Education	Right-Handed	MoCA	HAM-A	HAM-D
<5 years (*p*)	<0.001 ^χ2^	<0.001 ^KW^	0.019 ^KW^	0.209 ^KW^	<0.001 ^KW^	0.286 ^KW^	0.667 ^KW^
5–10 years (*p*)	0.012 ^χ2^	0.011 ^KW^	<0.001 ^KW^	0.008 ^KW^	0.002 ^KW^	0.004 ^KW^	0.004 ^KW^
>10 years (*p*)	0.221 ^χ2^	0.173 ^KW^	0.025 ^KW^	0.201 ^KW^	0.005 ^KW^	0.08 ^KW^	0.324 ^KW^
	**H&Y**	**SAS**	**Domain 3**	**Domain 5**	**Non-smoking**	**Cognitive impairment**	**Apathy**
<5 years (*p*)	<0.001 ^KW^	0.001 ^KW^	0.663 ^KW^	<0.001 ^KW^	0.528 ^KW^	<0.001 ^KW^	0.034 ^KW^
5–10 years (*p*)	<0.001 ^KW^	0.008 ^KW^	0.028 ^KW^	<0.001 ^KW^	0.011 ^KW^	0.031 ^KW^	0.006 ^KW^
>10 years (*p*)	0.167 ^KW^	0.002 ^KW^	0.115 ^KW^	0.03 ^KW^	0.333 ^KW^	0.917 ^KW^	0.343 ^KW^

^χ2^ = ^χ2^ test of independence, ^KW^ = Kruskal–Wallis test for independent samples.

## Data Availability

The data presented in this study are available on request from the corresponding author. Requests to access the datasets should be directed to Timotej Petrijan, timotej.petrijan@gmail.com.
